# 3D Automatic Segmentation of Aortic Computed Tomography Angiography Combining Multi-View 2D Convolutional Neural Networks

**DOI:** 10.1007/s13239-020-00481-z

**Published:** 2020-08-11

**Authors:** Alice Fantazzini, Mario Esposito, Alice Finotello, Ferdinando Auricchio, Bianca Pane, Curzio Basso, Giovanni Spinella, Michele Conti

**Affiliations:** 1 Department of Experimental Medicine, University of Genoa, Via Leon Battista Alberti, 2, 16132 Genoa, Italy; 2 Camelot Biomedical Systems S.r.l, Via Al Ponte Reale, 2, 16124 Genoa, Italy; 3 Department of Integrated Surgical and Diagnostic Sciences, University of Genoa, Genoa, Italy; 4 Department of Civil Engineering and Architecture, University of Pavia, Pavia, Italy; 5 Vascular and Endovascular Surgery Unit, IRCCS Ospedale Policlinico San Martino, University of Genoa, Genoa, Italy

**Keywords:** Aorta segmentation, Deep learning, Convolutional neural network, Multi-view integration

## Abstract

**Purpose:**

The quantitative analysis of contrast-enhanced Computed Tomography Angiography (CTA) is essential to assess aortic anatomy, identify pathologies, and perform preoperative planning in vascular surgery. To overcome the limitations given by manual and semi-automatic segmentation tools, we apply a deep learning-based pipeline to automatically segment the CTA scans of the aortic lumen, from the ascending aorta to the iliac arteries, accounting for 3D spatial coherence.

**Methods:**

A first convolutional neural network (CNN) is used to coarsely segment and locate the aorta in the whole sub-sampled CTA volume, then three single-view CNNs are used to effectively segment the aortic lumen from axial, sagittal, and coronal planes under higher resolution. Finally, the predictions of the three orthogonal networks are integrated to obtain a segmentation with spatial coherence.

**Results:**

The coarse segmentation performed to identify the aortic lumen achieved a Dice coefficient (DSC) of 0.92 ± 0.01. Single-view axial, sagittal, and coronal CNNs provided a DSC of 0.92 ± 0.02, 0.92 ± 0.04, and 0.91 ± 0.02, respectively. Multi-view integration provided a DSC of 0.93 ± 0.02 and an average surface distance of 0.80 ± 0.26 mm on a test set of 10 CTA scans. The generation of the ground truth dataset took about 150 h and the overall training process took 18 h. In prediction phase, the adopted pipeline takes around 25 ± 1 s to get the final segmentation.

**Conclusion:**

The achieved results show that the proposed pipeline can effectively localize and segment the aortic lumen in subjects with aneurysm.

**Electronic supplementary material:**

The online version of this article (10.1007/s13239-020-00481-z) contains supplementary material, which is available to authorized users.

## Introduction

Abdominal aortic aneurysm (AAA) is a vascular disease involving pathologic dilatations of the abdominal aorta up to more than 3 cm in the greatest diameter or dilatation of more than 50% of its diameter.^4^ The AAA is localized between the renal and iliac arteries and is associated with a high rate of morbidity and mortality.^15^

In the last few years, the surgical management of abdominal aortic aneurysms has shifted from surgery to minimally invasive endovascular repair (EVAR). During the intervention, the surgeon deploys one or more stent grafts into the aneurysm sac using a catheter inserted through femoral access: this procedure reduces the pressurization of the aneurysm sac lowering the risk of wall rupture.^8^

Computed Tomography Angiography (CTA) is the primary imaging technique used to assess, manage, and monitor abdominal aortic aneurysms. Accurate segmentation of the aortic lumen in CTA scans is a critical step to measure aortic length and diameters in order to facilitate the sizing of endografts.^2^ Besides the clinical applications, the aortic models obtained from medical images are also exploited to perform numerical simulations.^16^

However, commercial software are semi-automatic and require user initialization to perform vessel morphological analysis.^6^ An automatic tool performing aortic lumen segmentation would facilitate and standardize the analysis of the aortic anatomy, enabling robust and reproducible measurements.

Recently, deep learning techniques have shown excellent performances in the field of medical image analysis, addressing classification, segmentation, and detection tasks.^18^ In the following, some studies showing the potential of deep learning-based techniques in the endovascular field are reported.

Larsson *et al.* proposed a fully automatic method to perform the segmentation of abdominal organs including abdominal aorta.^7^ A feature-based multi-atlas approach is exploited to coarsely localize the organ, then a 3D Convolutional Neural Network (CNN) is used to perform segmentation. In this study, the overall dataset included 70 CT images of abdominal organs.

Lopez *et al.* designed a fully automatic pipeline performing thrombus detection and its subsequent segmentation.^9^ Thrombus segmentation is performed on single 2D slices, and the spatial consistency between sequential slices is enforced at a second stage applying a Gaussian filter in the *z*-direction; the dataset is composed of 13 postoperative CTA. This work has been subsequently extended using 3D networks instead of 2D networks^8^

Noothout *et al.* suggested an automatic method to segment the ascending aorta, the aortic arch and descending aorta in low-dose, non-contrast-enhanced scans.^14^ A dilated convolutional neural network is applied to axial, coronal, and sagittal planes, then the results obtained in each view are averaged to provide the final segmentation. The dataset is composed of 24 CT scans.

In the work by Mohammadi *et al.*, the aorta is detected in CTA scans using a patch-wise CNN classifier.^13^ After detection, the aorta borders are extracted through the Hough Circle algorithm and the lumen diameters are used to predict the risk of AAA. The dataset used to train the CNN classifier is composed of 5800 image patches.

Few studies have been carried out for the automatic segmentation of aortic lumen in type B aortic dissection. In Cao *et al.,* multitask learning is exploited to segment the whole aorta, the true lumen and the false lumen using 3D CNNs.^1^ The dataset is composed of 276 CTA scans.

Despite some works based on deep learning have been proposed already to perform detection and segmentation of aortic lumen and thrombi, most of them are based on 2D CNNs and do not deal with 3D spatial coherence, targeting for specific segments of the aortic lumen (thoracic, abdominal, or iliac) or thrombosis. To overcome such limitations, the present study develops a deep learning approach aimed at locating and segmenting the whole aortic lumen from thoracic aorta to the common iliac arteries, including branch vessels in the aortic arch and abdominal segment, accounting for spatial consistency. In particular, we have implemented a fully automatic pipeline for preoperative aortic lumen segmentation, relying on a first CNN to coarsely segment and locate the aortic lumen from the whole CTA scans followed by other CNNs for its finer segmentation.

Since deep learning approaches are data and memory demanding, the whole pipeline exploits 2D CNNs instead of 3D CNNs.

## Materials and Methods

**Figure 1:**
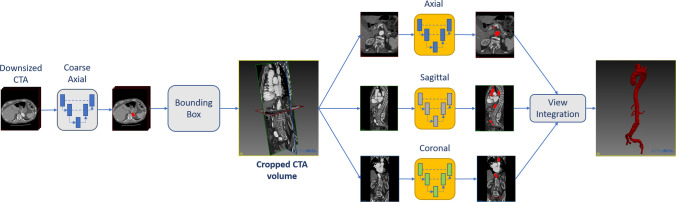
Proposed method for aorta segmentation. On the left, the coarse localization of aorta is performed on down-sampled images. On the right, models trained on axial, sagittal and coronal axes are used to perform single-view segmentations from the cropped CTA volume. The final segmentation is obtained by integrating the three orthogonal segmentations.

The proposed approach for automatic aortic segmentation is described in Fig. 1. The pipeline consists of a first 2D U-Net trained on down-sampled (¼) axial slices in order to localize and extract a preliminary aortic mask from CTA scans. The extraction of a Region of Interest (ROI) is performed both to reduce the needed memory and to focus on the information that is crucial to segment the aorta. The identified ROI is then processed by two-dimensional U-Nets trained on axial, sagittal, and coronal planes that are obtained extracting 2D slices along the x, y, and z axes of the CTA scan under higher resolution. The predictions provided by the three planes U-Nets are then combined to provide a final segmentation that is spatially coherent, overcoming the limitations of single-plane CNNs.

### Dataset

The dataset used in this study has been provided by IRCCS San Martino University Hospital (Genoa, Italy) and consists of 80 preoperative Computed Tomography Angiography (CTA) scans regarding patients with abdominal aortic aneurysm as primary pathology. Patients with other diseases such as aortic dissections and thoracic aneurysms were not included.

All the subjects involved in the study have signed the informed consent form, allowing the treatment of their anonymized data for research purposes. Given the retrospective nature of the analysis and the adoption of anonymized data, we have not submitted the ethics committee/IRB application.

The mean age of the patients was 75 years (range 60–91 years), with a male predominance (86% males compared to 14% females). Each CTA volume has been semi-automatically segmented by trained experts by means of ITK-Snap interactive tool.^19^ The ground truth segmentations include thoracic, abdominal, and iliac aortic segments. In addition, aortic arch branch vessels (innominate artery, left common carotid artery, and left subclavian artery) and abdominal aortic branch vessels (celiac trunk, superior mesenteric artery, left and right renal arteries) were included in the segmentations. The branch vessels were segmented up to a distance of 2 cm from the beginning of the branch. The CTA acquisitions and corresponding segmentations have been divided into three groups: training set (*n* = 64 scans), validation set (*n* = 6 scans), and test set (*n* = 10 scans). While the train and the test sets are respectively exploited to train and evaluate the network, the validation set is used in three different ways. During the training process, the validation set is employed to prevent data overfitting through early stopping regularization. Besides regularization, the validation set is used to define the threshold needed to binarize the raw probability maps provided by the network. Finally, the validation set is exploited to select the integration approach to combine the single-axis predictions.

### Data Preprocessing

Data preprocessing is crucial to properly train the deep learning networks; in the following, all the steps involved in data preprocessing are described.

- Voxel size normalization: since input scans have varying spatial resolution (pixel size 0.76 ± 0.08 mm, spatial thickness 0.75 ± 0.26 mm), the CTA pixel size has been set to 0.73 mm and the slice thickness to 0.62 mm to enable the network to properly learn the spatial semantic. The selected pixel size and slice thickness are computed as the median pixel size and thickness in the dataset.
- Window level adjustment: to enhance the aortic lumen in the CTA scans, the window level has been set to 200 Hounsfield Units (HU) with 800 HU window width, resizing the image intensity between 0 and 255.
- Resizing: in order to train the first U-Net and coarsely extract the aorta location, the axial slices have been down-sampled to 128x128 pixels (with down-sample factor = 4).
- ROI Extraction: given the preliminary axial segmentation, a cuboid of dimension 144 × 144 × 480 centered in the aorta is used to crop the CTA images at higher resolution (down-sample factor = 2) and exclude part of the background. The cuboid is used to train both axial, sagittal, and coronal U-Nets.
- Data Augmentation: random rotations, shifts, and zoom factors are applied to the slices during the training process to augment both the training and the validation sets (Table 1).

**Table 1:** Data augmentation parameters.

Methods	Coarse segmentation	Finer axial segmentation	Finer sagittal and coronal segmentation
Degree range for rotations	[− 10°, 10°]	[− 7°, 7°]	[− 7°, 7°]
Width shift range	[− 20, 20] pixels	[− 22, 22] pixels	[− 22, 22] pixels
Height shift range	[− 22, 22] pixels	[− 22, 22] pixels	[− 72, 72] pixels
Zoom range	[0.85, 1.15]	[0.85, 1.15]	[0.85, 1.15]

### Pipeline

Based on the proposed workflow, this subsection is divided into three main steps such as (a) aortic lumen localization, (b) single-view segmentation, and (c) multi-view aggregation.

- Localization. Segmentation is considered as a binary classification problem, thus each pixel in the image is classified as aorta or background type. A preliminary 2D U-Net model is used to segment the aorta from the axial view. As already reported in the previous section, the network is trained and validated on CTA images resized to [128, 128] to reduce the needed memory. The probability map provided by the 2D coarse model is binarized with a single threshold that maximizes the Dice Coefficient (DSC) on the validation set. Then, the coarse segmentation is used to extract a bounding box of the aortic mask and keep only the area around the aorta. The bounding box coordinates are used to get the ROI with higher resolution, as proposed by Jia *et al.*^3^
- Single-view Segmentation. Each CTA scan is parsed into 2D axial, sagittal, and coronal views. The scans are cropped with the cuboid computed in the previous step. The cropped scans have half resolution with respect to the original CTA scans to deal with memory needs. According to Noothout *et al.*, a separate deep learning model is trained for each orthogonal view using the same U-Net architecture.^14^ The three networks are trained using the same CTA scans, but taken from different perspectives.
- Multi-view Aggregation. Three different U-Nets have been separately trained on axial, sagittal, and coronal planes to deal with the need for spatial coherence in segmentation. The outputs provided by the single-plane networks are 2D probability maps where each intensity value represents the probability of a given pixel being aortic lumen or not. According to Lopez *et al.*, each 3D prediction map volume is obtained by concatenating the 2D probability maps and applying a Gaussian filter along the z-direction.^9^ Similar to the coarse segmentation, the single-view 3D label map is obtained by binarizing the prediction map with a threshold maximizing the DSC on the validation set. The aggregation stage is intended to regularize the voxel prediction by considering the spatial information from the three orthogonal views. Three different approaches have been implemented to combine the single-view segmentations:
  Majority voting. Each network makes a prediction for the voxels in the CTA scan, and the corresponding label maps are generated. According to the approach followed by Zhou *et al.*, the predicted label (aorta or background) is assigned to the voxel following the majority voting rule among the predictions of the single-view networks.^20^
  Simple averaging. According to Noothout *et al.,* the single-view prediction maps are averaged to provide a final prediction map.^14^ The final prediction for each voxel $x$ in the per-view prediction maps is computed as follows:
    $$p_{\text{final}} (x) = \frac{1}{3} p_{\text{ax}} (x) + \frac{1}{3} p_{\text{sag}} (x) + \frac{1}{3} p_{\text{cor}} (x)$$
    where $p_{\text{ax}} (x)$, $p_{\text{sag}} (x)$, $p_{\text{cor}} (x)$ are the voxel prediction in axial, sagittal, and coronal views respectively. The binary segmentation is finally obtained by thresholding the obtained prediction map.
  Integration of majority voting and averaging. Given the 3D label maps, if the three networks agree on the same label (aorta or background), the probability value in the final probability map is set to be 1 if the label is aorta, or 0 if the label is the background. Otherwise, the final voxel probability is averaged following the previous formula, as proposed by Lyksborg *et al.*^10^ The binary segmentation is finally obtained by thresholding the obtained prediction map.

### Data Analysis

The segmentations obtained with the proposed pipeline are compared against the ground truth annotations using multiple criteria.

- Overlap measures. The Dice coefficient is used to evaluate the performance of the multi-view network as an overlap measure between the predicted and the ground truth segmentations. The Dice coefficient between two binary segmentations is defined as follow:
  $${\text{DSC}} = \frac{{2 |{\text{GT}} \cap P|}}{{|{\text{GT}}| + |P|}}$$
  where GT is the ground truth volume and P is the automatically segmented volume. The Jaccard index (JAC), defined as intersection between the two volumes divided by their union, is further considered:
  $$JAC = \frac{{\left| {GT \cap P} \right|}}{{\left| {GT \cup P} \right|}}$$
- Symmetric surface to surface distance. This criterion is used to evaluate how closely the surfaces generated by the predicted and the ground truth segmentations align. The distances are obtained by means of the distance maps proposed by Maurer *et al.*^12^ Mean and standard deviations of the surface-to-surface distances are used to represent how the two surfaces globally align, while the maximum distance is used to represent the spurious errors.

## Experiments and Results

### Experimental Settings

The experiments were performed using 80 CTA scans acquired in the same hospital. Both training, validation and testing were performed on a NVIDIA GeForce RTX 2080 Ti graphic card with CUDA compute capability = 7.5, under Windows operating system. The segmentation pipeline was completely developed using Python. The deep learning models were implemented in Keras framework based on Tensorflow with GPU support.

To train the models, binary cross-entropy was used as a loss function, and Adam optimizer with learning rate = 0.0001 was adopted to optimize the network parameters. In each iteration, a mini-batch containing 20 and 10 slices randomly sampled from the training set was provided to the first U-Net and to the single-view networks, respectively. The images in the mini-batch were modified on the fly with random rotations, shifts, and zoom factors (Table 1) to perform data augmentation. The training process was stopped using early stopping criteria, with patience set to 15 epochs. The overall time needed to train the first U-Net and the single-view U-Nets are reported in Tables 2 and 3, respectively.

**Table 2:** A summary of data employed to perform coarse axial segmentation together with information on the training process is reported in the first columns.

Segmentation task	Training CTA	Validation CTA	Test CTA	Image dimension	Number of epochs	Training time (min)	Binarization threshold	DSC on test set	DSC on validation set
Axial coarse segmentation	64 (61507 2D slices)	6 (5795 2D slices)	10 (9033 2D slices)	(128, 128)	19	111	0.4	0.92 ± 0.01	0.92 ± 0.03

The quantitative results obtained on the test set and validation set are shown in the last columns on the right. The reported binarization threshold maximizes the DSC on the validation set

**Table 3:** A summary of data employed to perform single-view segmentation along with information on the training process is reported in the first columns.

Segmentation task	Training CTA	Validation CTA	Test CTA	Image dimension	Number of epochs	Training time (min)	Binarization threshold	Dice coefficient
Axial	64 (47087 2D slices)	6 (4744 2D slices)	10 (7411 2D slices)	(144, 144)	18	252	0.4	0.920 ± 0.016
Sagittal	64 (11658 2D slices)	6 (1063 2D slices)	10 (1727 2D slices)	(480, 144)	35	378	0.45	0.915 ± 0.038
Coronal	64 (11122 2D slices)	6 (1113 2D slices)	10 (1775 2D slices)	(480, 144)	36	327	0.4	0.913 ± 0.019

The quantitative results obtained on the test set are shown in the last column on the right. The reported binarization threshold maximizes the DSC on the validation set

### Aortic Lumen Coarse Segmentation and Localization

Since the first U-Net is just aimed at coarsely identifying the aortic lumen in the CTA scans, the performances were evaluated only in DSC terms, regardless of Jaccard score and surface to surface distances. Table 2 lists the average ± standard deviation DSC achieved on the 10 test scans.

### Single-Plane Lumen Segmentation

Axial, sagittal, and coronal U-Nets were separately trained on the CTA cropped around the area of interest. Table 3 reports the average ± standard deviation Dice coefficients achieved on the 10 test scans.

### Multi-view Aggregation

Multi-view aggregation is the final step in the proposed pipeline. Three different approaches were implemented to integrate axial, sagittal, and coronal predictions into a final segmentation. The evaluation of the integration approaches was performed on the validation set. As shown in Table 4, the three approaches provide similar results in terms of overlap and surface measures. Simple averaging and the combination of majority voting and averaging provide the same results to the fifth decimal place. Since the combination of majority voting and averaging provided shade better results, it was selected as the final integration approach.

**Table 4:** Performances of different multi-view aggregation approaches on the validation set.

Integration approach	Dice coefficient	Jaccard coefficient	Mean surface distance (mm)	Maximum distance (mm)
Majority voting	0.929 ± 0.021	0.869 ± 0.037	0.575 ± 0.193	32.888 ± 10.17
Simple averaging	0.930 ± 0.021	0.870 ± 0.036	0.559 ± 0.188	28.020 ± 5.807
Integration of majority voting and averaging	0.930 ± 0.021	0.870 ± 0.036	0.559 ± 0.188	28.020 ± 5.807

Both Dice and Jaccard scores and mean and maximum symmetric surface distances are evaluated

The results obtained with multi-view aggregation were compared with those provided by single-view segmentations to evaluate the impact of the proposed approach on the test set. As it can be noticed from Table 5, combining orthogonal segmentations provided better results than those obtained with single view segmentations.

**Table 5:** Performances provided by single-view and multi-view segmentations on the test set.

View	Dice coefficient	Jaccard coefficient	Mean surface distance (mm)	Maximum distance (mm)
Axial	0.920 ± 0.016	0.853 ± 0.02	1.457 ± 0.695	121.772 ± 27.356
Sagittal	0.915 ± 0.038	0.845 ± 0.060	2.451 ± 3.680	75.073 ± 42.470
Coronal	0.913 ± 0.019	0.841 ± 0.032	1.363 ± 1.179	82.364 ± 31.086
Multi-view integration	0.928 ± 0.013	0.866 ± 0.023	0.711 ± 0.644	50.059 ± 37.285

Both Dice and Jaccard scores and mean and maximum symmetric surface distances are evaluated

**Figure 2:**
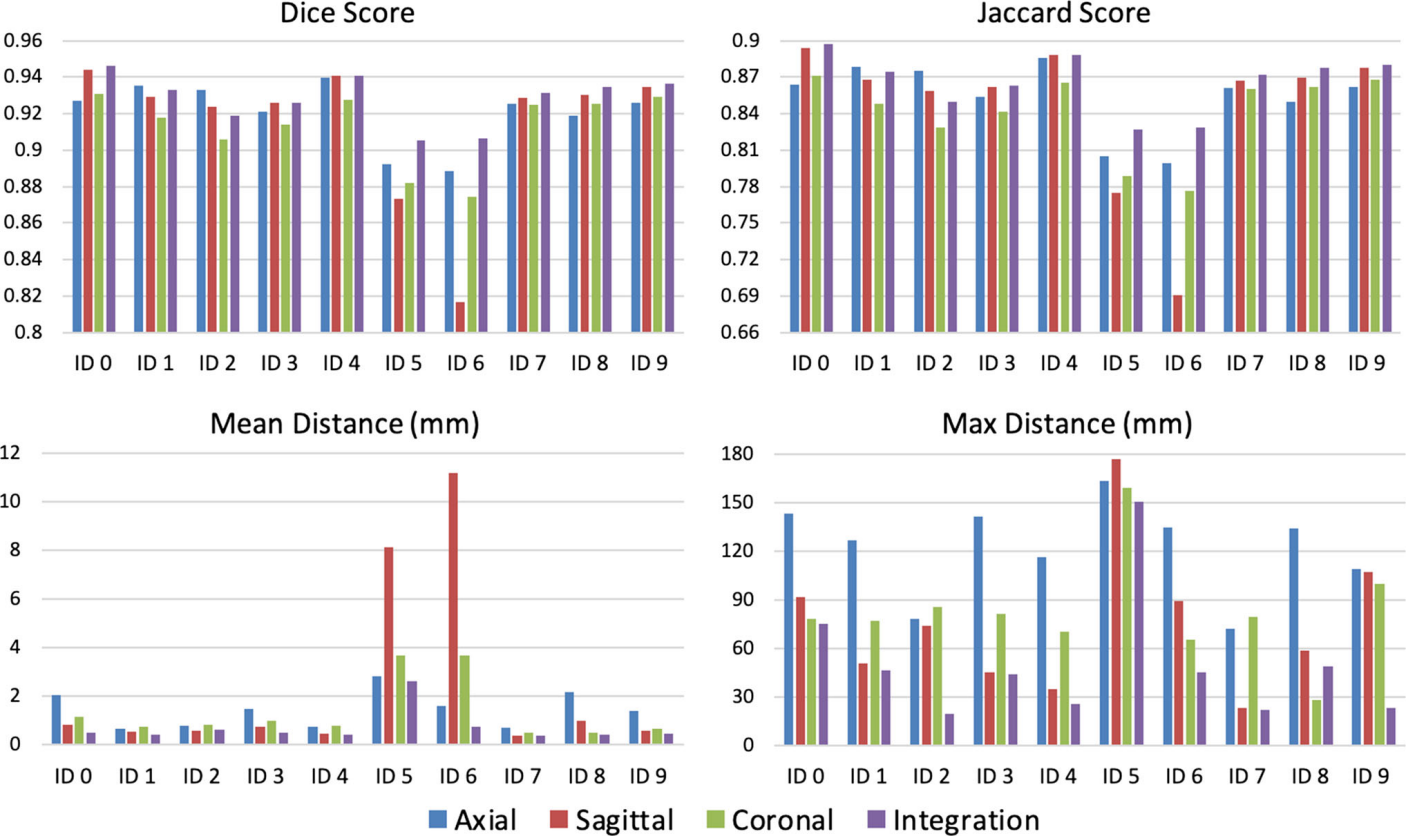
The Dice and Jaccard scores, together with mean and maximum distance measurements, are reported for each patient in the test set. Each patient is represented by an identification number (ID). For each evaluation metric, the results provided by single-view and multi-view segmentation are reported.

**Figure 3:**
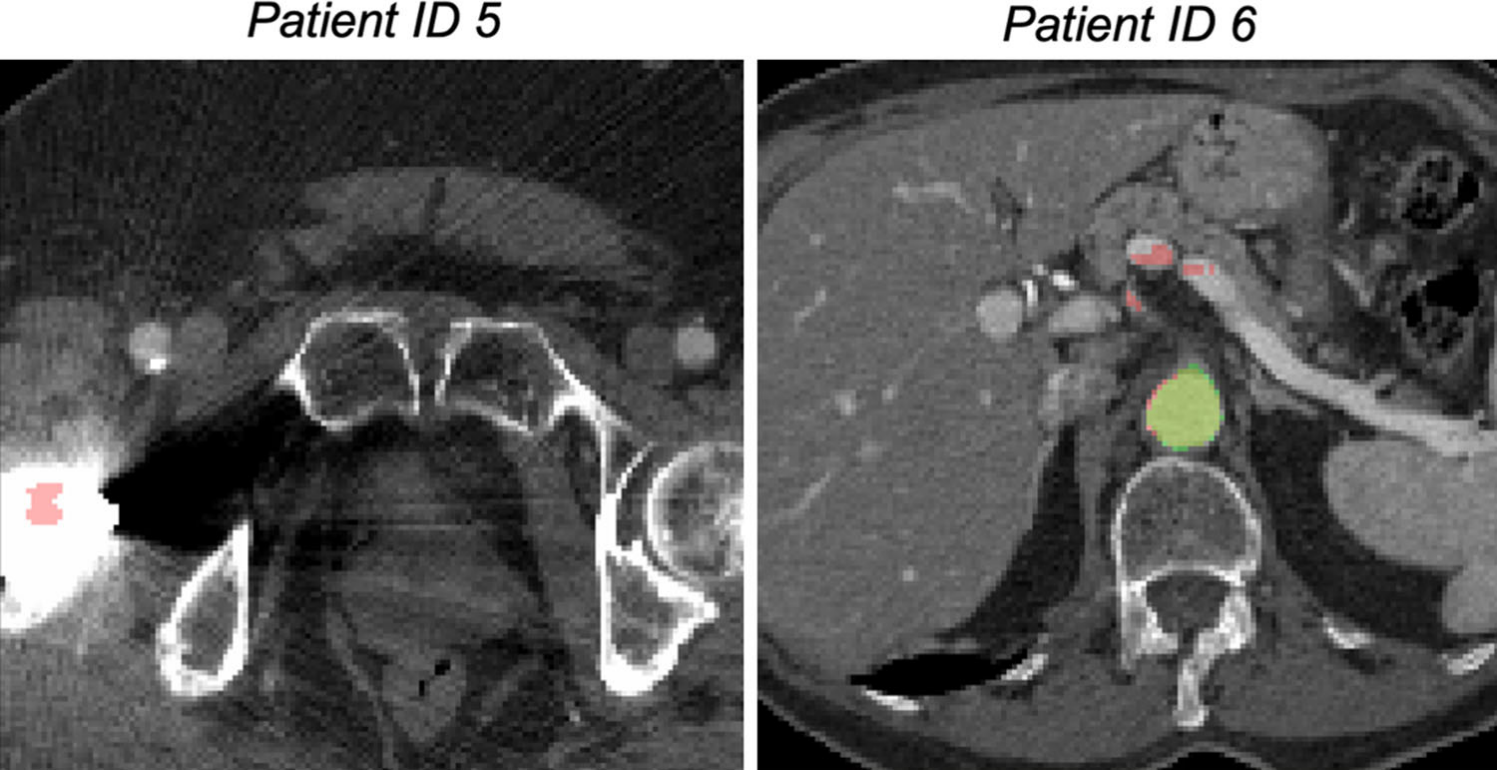
Patient with ID5 presents a high maximum surface-to-surface distance because some pixels are wrongly labeled as aorta in the iliac area. The presence of an orthopedic prosthetic may have caused these errors. In Patient with ID6, some spurious errors are caused by the partial segmentation of other vessels near the aorta.

The quantitative measures of performance achieved with the single-view and the multi-view models on individual test patients are reported in Fig. 2. Patients with ID5 and ID6 present the lowest DSC and Jaccard index as well as the higher mean and maximum surface-to-surface distances. Patient with ID5 has an orthopedic prosthesis that caused artifacts in the acquired CTA. The high maximum surface-to-surface distance is caused by the fact that part of the prosthesis has been segmented as part of the aorta (Fig. 3). As regards the patient with ID6, we have noticed that parts of vessels near the aorta have been included in the predicted segmentation, increasing the number of false positives. Probably, the network segments parts of those vessels as they have similar features to those of the aorta and the other branches included in the ground truth segmentations (Fig. 3).

Increasing the training set may reduce the errors highlighted for patients with ID5 and ID6. Including more patients with orthopedic prosthesis may avoid the errors encountered in ID5 segmentations, reducing the number of false positives. Besides, the inclusion of CTAs with heterogeneous anatomies may increase the network ability to correctly identify the aortic lumen making the segmentation more robust.

**Figure 4:**
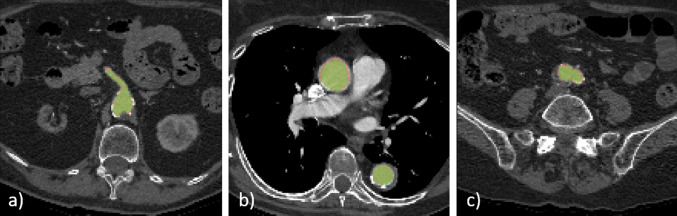
Ground truth and predicted segmentations are overlaid onto a cropped CTA belonging to the test set. The ground truth segmentation is represented in green, while automatic segmentations is displayed in red.

**Figure 5:**
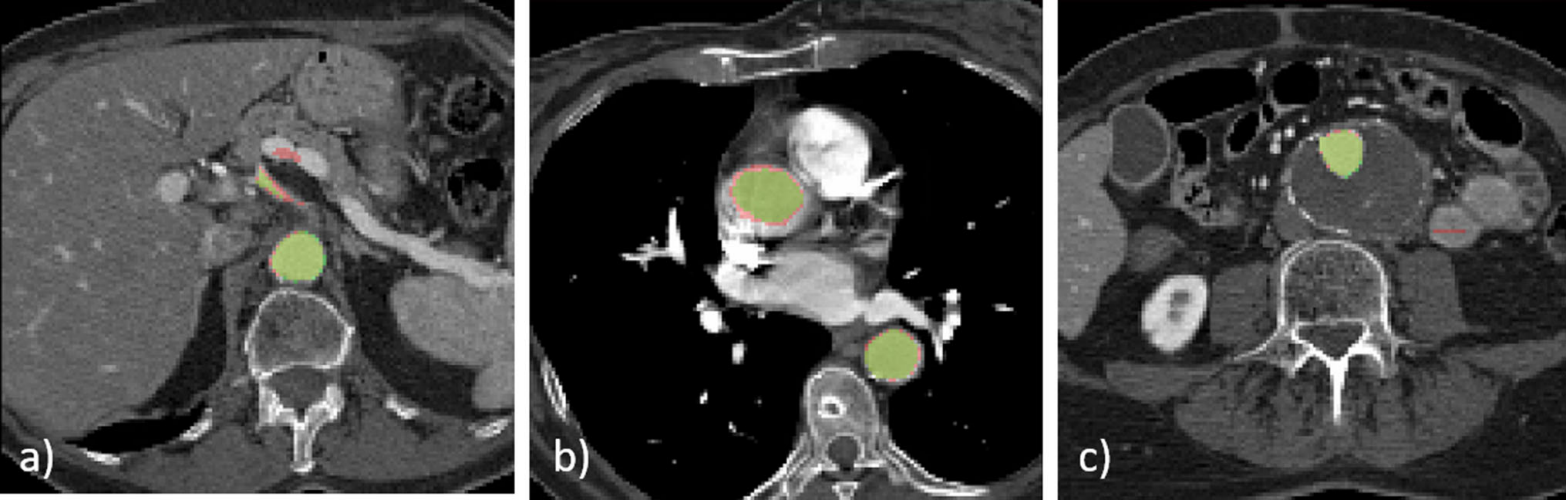
Ground truth and predicted segmentations are overlaid onto a cropped CTA belonging to the test set. The ground truth segmentation is represented in green, while automatic segmentations is displayed in red. The automatic segmentation in slices (a) and (c) presents some spurious errors.

A qualitative evaluation of the results provided by the developed pipeline is presented in Figs. 4 and 5, where the aortic segmentations performed by the experts are overlaid with the segmentations obtained with multi-view aggregation. In Fig. 4, the segmentation provided by the proposed method is smooth and effective. In Fig. 5, the final result presents some spurious errors.

**Figure 6:**
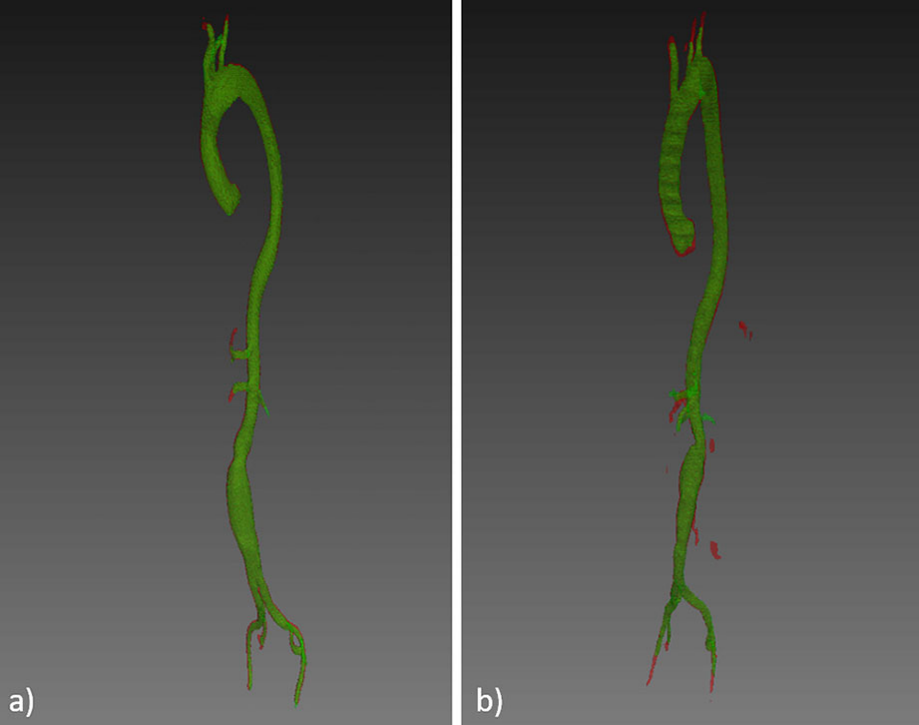
The ground truth 3d model and the model generated by automatic CTAs segmentation are represented in green and red, respectively. The considered CTAs belong to the test set. In panel (a) the predicted model aligns with the ground truth. In panel (b), the predicted model presents some spurious areas and extends deeper into the iliac arteries.

Figure 6 presents the 3D models generated from the automatic and ground truth segmentations represented in Figs. 4 and 5.

## Discussions and Conclusion

In this study we have proposed a deep learning approach for a spatially coherent segmentation of thoracic aorta, abdominal aorta, and iliac arteries.

In the proposed pipeline, the aortic lumen is first coarsely localized in the CTA scans, then the axial, sagittal, and coronal slices are segmented from the cropped region of interest using three distinct 2D networks. Finally, the single view segmentations are combined to provide the final segmentation. The presented method can localize and effectively segment the aorta from preoperative CTA scans of patients affected by AAA.

Since the lack of annotated data is one of the most common limitations encountered in many deep learning approaches,^8^, ^9^, ^14^ a semi-automatic segmentation protocol was established to collect a labeled dataset. The experts have segmented a total amount of 80 CTA volumes to enable end-to-end learning. Since each CTA scan is decomposed into stacks of axial, sagittal, and coronal 2D slices, the networks were trained with a large amount of data.

Moreover, the CTAs in our dataset comprise annotations of thoracic, abdominal, and iliac segments, including aortic arch branch vessels (innominate artery, left common carotid artery, and left subclavian artery) and abdominal aortic branch vessels (celiac trunk, superior mesenteric artery, left and right renal arteries), while most of the studies available in literature are focused on a single area.^7^–^9^, ^14^ In this way, the proposed approach allows the analysis of the whole aortic morphology.

### Axial Pre-segmentation

The first step in the proposed pipeline is the automatic, coarse identification of the aortic lumen from CTA scans. This step is performed to preserve only the information needed to segment the aortic lumen, excluding part of the background. In the work proposed by Lopez *et al.*, a 2D specific detection network is exploited to perform thrombus localization from CTA images.^9^ In our work, we have adopted another approach exploited for left atrium automatic segmentation.^3^ Here, a first 3D U-Net was exploited to extract the region of interest from MR images, then a second 3D U-Net performed a finer segmentation on MR cropped at higher resolution.

### Spatial Coherence

In our work, three state-of-the-art approaches performing multi-view integration were evaluated to find out which one suits our problem better; our results showed that the integration of majority voting with simple averaging provided the best prediction.

Since 3D CNNs usually require larger dataset and are memory-demanding,^5^ some studies focused on the integration of orthogonal 2D-CNNs for medical image segmentation.^10^, ^11^, ^17^, ^20^ To the best of our knowledge, only one publication deals with 3D integration of 2D CNNs in the endovascular field,^14^ where the authors average together axial, sagittal, and coronal predictions to provide a smoother segmentation of the aortic lumen from CT scans. Following these state-of-the-art approaches, we have adopted a combination of 2D U-Nets trained on orthogonal planes to provide a final 3D segmentation.

### Limitations

- *Dataset* A larger dataset should further improve the performances of the proposed pipeline, leading to a better generalization and preventing overfitting problems. Moreover, including CTA acquired in different hospitals and with different scanners should increase the variability in the training, validation and test sets, leading to better segmentation results.
- *Segmentation accuracy* In order to train the deep learning models involved in the adopted pipeline, a dataset composed of 80 CTAs has been semi-automatically segmented by trained experts. The models in the pipeline learn in a supervised manner how to segment CTA images. As a consequence, the accuracy of the pipeline strongly depends on the quality of the ground truth segmentations generated by the experts using the semi-automatic software. Having the same CTAs segmented by several experts would be helpful to deal with interobserver variability. However, since ground truth generation is a time-consuming task, in the present study we have provided only one ground truth segmentation for each patient.
- *Lumen segmentation* In this work, the proposed pipeline segments the aortic lumen from CTAs, without considering the thrombus. Currently, the lack of annotated thrombi in our CTA scans prevented the development of this line of research. Moreover, since multi-class segmentation is more complex than binary segmentation, a larger dataset could be required to train the multi-class networks. In future works we aim at integrating the thrombus segmentation part.
- *Preoperative CTA segmentation* Another limitation of the current work is that the proposed pipeline is limited to preoperative CTA scans. It would be very interesting to perform automatic segmentation of both preoperative and postoperative aortic lumen, in order to evaluate the morphology changes after EVAR. In future works, after collecting and labeling postoperative CTAs, the developed pipeline will be trained and tested on postoperative data.
- *Patient anatomy* In this work, we have not analyzed the impact of patient anatomy on the segmentation results. In future developments, given a bigger test set it might be possible to evaluate the variation of the segmentation performance with respect to the patient anatomy. This analysis might enable the identification of anatomies that are more difficult to segment and the development of a classification algorithm capable of automatically identifying these challenging cases.
- *ITK-Snap plugin* Currently, the developed pipeline is not integrated in ITK-Snap. Further developments of the present study will address the implementation of ITK-Snap plugins to make the developed pipeline available.

### Conclusion

The proposed automatic segmentation pipeline shows promising applications both for clinical practice and numerical analysis.

Considering that the aortic lumen segmentation is the first step for morphology analysis and stent-graft sizing, this approach may significantly reduce the workload for surgeons in the planning stage and facilitate decision making. Moreover, the automatic segmentation of the lumen in all the aortic segments represents an advantage, as it enables different types of geometric analysis (e.g., iliac tortuosity estimation, thoracic aortic arch analysis, *etc*.).

Finally, the 3D models obtained from the automatic segmentation may be used to perform different types of numerical analysis (e.g., simulation of guidewire insertion during EVAR, simulation of stent graft deployment, hemodynamic simulations, *etc*.).

On average, the whole pipeline took only 25 ± 1 s per scan, making it suitable for application in studies including large volumes of images.

## Electronic supplementary material

Below is the link to the electronic supplementary material.

Supplementary material 1 (DOCX 109 kb)
