# Supplementary material for: 3D Automatic Segmentation of Aortic Computed Tomography Angiography Combining Multi-View 2D Convolutional Neural Networks
Source: Cardiovasc Eng Technol. 2020 Aug 11;11(5):576–86. doi: 10.1007/s13239-020-00481-z (PMC7511465; doi:10.1007/s13239-020-00481-z)
Supplement: Supplementary file 1 — Supplementary material 1 (DOCX 109 kb) [file 13239_2020_481_MOESM1_ESM.docx]

# 5. Supplementary Material

**5.1 U-Net architecture**

U-Net is a Convolutional Neural Network designed for fast and precise segmentation of biomedical images [1]. The architecture consists of a contracting path and an expansive path (**Fig 7**).


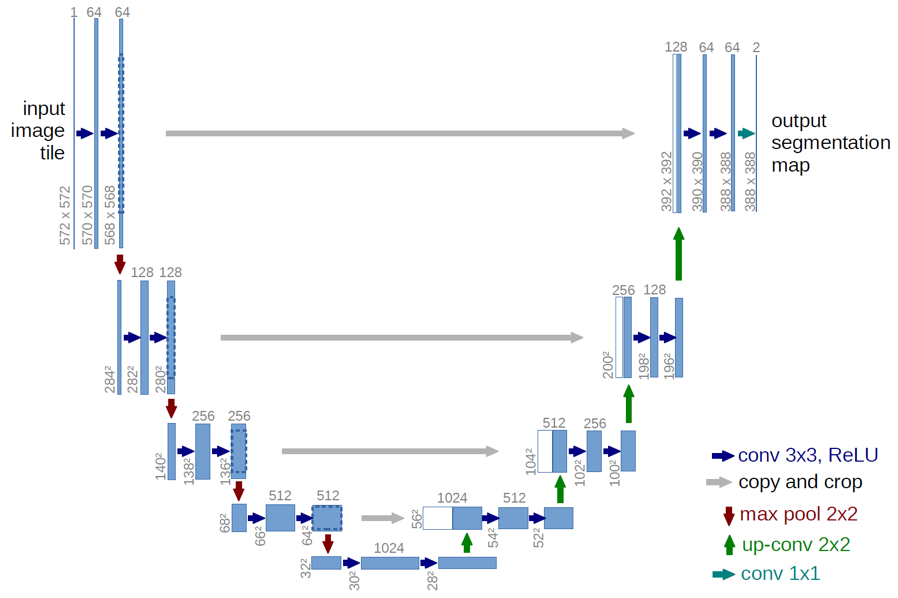


**Fig 7** *U-Net architecture as proposed in [1]*

The contractive path on the left is intended to capture context and it follows the typical architecture of a convolutional network. It consists of many contraction blocks composed of two convolutions each followed by a rectified linear unit (ReLU) and a max pooling operation. The expansive path on the right is intended to enable precise localization. It consists of many expansion blocks composed by up-convolution, a concatenation with the corresponding feature map in the contractive path, and two convolutions each followed by a ReLU.

We have followed U-Net architecture and added a Batch Normalization layer before the activation function.

**5.2 U-Net training**

The input CTAs and their corresponding ground truth segmentations are used to train the network with mini-batch gradient descent implementation on Keras framework. Adam optimizer with learning rate = 0.0001 was adopted to optimize the network parameters. Binary cross-entropy was used as loss function. This loss evaluates each pixel individually by comparing the output predicted by the network to the ground truth label.

For each pixel in the segmentation mask, the loss can be defined as follows:

$$Loss= -(ylog\left( p \right)+\left( 1-y \right)log(1-p))$$

with $y$ the pixel label (0 = background; 1 = aorta) and $p$ the pixel probability of being part of the aorta.

In order to reduce data overfitting during the training process, both dropout and early stopping have been exploited as regularization techniques. To implement the early stopping strategy, we have evaluated the loss on the validation set at the end of each training epoch. The training process is stopped after 15 epochs (e.g., *patience* = 15) with no progress on the validation set. Then, the model with the lowest error on the validation set is kept as the final model.

**5.3 Additional evaluations**

Since segmentation can be seen as a pixel-wise classification problem, confusion matrix can be used to evaluate the performance of the segmentation pipeline. The matrix allows the evaluation of true positives, true negatives, false positive and false negatives.
Some additional metrics have been computed from the confusion matrix:

- **Error:** fraction of predictions the model got wrong
- **Accuracy:** fraction of predictions the model got right
- **Sensitivity (recall):** probability that the model classifies the pixel as being part of the aorta given that it belongs to the aorta.
- **Precision:** proportion of pixels that are correctly labelled as aorta over the whole pixels the model labels as aorta.
- **Specificity:** probability that the model classifies the pixel as being part of the background given that it belongs to the background.

The segmentations obtained with multi-view integration are compared to ground truth segmentations using a confusion matrix (**Table 6**). Given the confusion matrix, some metrics have been computed (**Table 7).** Precision is the lowest of all the metrics obtained from the confusion matrix as some segmentations present spurious errors.

**Table 6** *Confusion matrix computed on the test set.*

|  |  | **Predicted label** | |
| --- | --- | --- | --- |
|  |  | **Negative** | **Positive** |
| **Observed label** | **Negative** | 196390698 | 308243 |
|  | **Positive** | 46798 | 2319861 |

**Table 7** *Metrics computed from the confusion matrix.*

| **Error** | **Accuracy** | **Sensitivity (Recall)** | **Precision** | **Specificity** |
| --- | --- | --- | --- | --- |
| 0.002 | 0.998 | 0.980 | 0.882 | 0.998 |

**References**

[1] O. Ronneberger, P. Fischer, and T. Brox, “U-Net: Convolutional Networks for Biomedical Image Segmentation,” *ArXiv150504597 Cs*, May 2015, Accessed: Jun. 10, 2020. [Online]. Available: http://arxiv.org/abs/1505.04597.
